# Metabolomics in cancer detection: A review of techniques, biomarkers, and clinical utility

**DOI:** 10.37796/2211-8039.1665

**Published:** 2025-09-01

**Authors:** Grisilda Vidya Bernhardt, Kavitha Liegelin Bernhardt, Janita R.T. Pinto, Asha Vashe

**Affiliations:** a Department of Biochemistry, RAKCOMS, Ras Al-Khaimah Medical and Health Sciences University, Ras Al-Khaimah, United Arab Emirates; b Division of Physiology, Department of Basic Medical Sciences, Manipal Academy of Higher Education, Manipal, Karnataka, India; c Department of Biomedical Sciences, College of Medicine, Gulf Medical University, Ajman, United Arab Emirates

**Keywords:** Metabolomics, Early cancer detection, Non-invasive tests, Biomarkers, Computational tools

## Abstract

Cancer poses a significant burden on global public health, contributing to high mortality rates worldwide. Ongoing diagnostic strategies have predominantly relied on imaging techniques, histopathological examination and molecular analyses which have limitations in sensitivity, and specificity. Early cancer detection is a pivotal determinant of successful treatment and patient survival rates. Metabolomic applications involve the comprehensive analysis of metabolites to understand the metabolic profile of an organism, tissue, or cell under different conditions such as lack of oxygen in tumors. The aim of this review is to provide an extensive approach of metabolomic applications in early cancer detection and to provide an overview of the strengths and limitations of metabolomic approaches in early cancer detection. Metabolomic profiling can identify specific metabolic biomarkers indicative of early-stage cancer. The identification of these biomarkers can lead to development of non-invasive diagnostic tests which can be used for early cancer screening. Several researchers have already employed the metabolomics approach for biomarker discovery, diagnosis, identifying new drug targets along with the clinical trials observations. When discussing challenges, researchers currently face a notable obstacle, the absence of standardized analytical procedures. It is imperative for the field to prioritize implementing computational tools for constructing open-source databases, thereby advancing metabolomic studies in cancer research.

## 1. Introduction

Timely detection of cancer often expands therapeutic options. In its early stages, cancer is typically localized and confined to a specific area, making it more amenable to treatment through surgery, radiation therapy, or other targeted modalities. However, as the disease progresses, metastasis can occur, complicating treatment and reducing the likelihood of favorable outcomes [1]. To mitigate cancer-related mortality on a global scale, there is an urgent need for advanced diagnostic technologies capable of accurately identifying the location, size, stage, and molecular characteristics of tumors. Current diagnostic approaches encompass a combination of physical examinations, laboratory investigations (e.g., blood and urine tests), and non-invasive imaging techniques such as computed tomography (CT), ultrasonography, magnetic resonance imaging (MRI), and bone scans. These are often supplemented by invasive procedures, including needle aspirations, surgical biopsies, and other specialized techniques to determine cancer type and stage [1–4].

This review provides a comprehensive overview of the current landscape of research, challenges, and future directions in leveraging metabolomics for cancer diagnosis. It highlights the potential of metabolomics to identify disease-specific biomarkers that enable early-stage detection, offering a promising avenue to improve patient outcomes.

## 2. Current applications and methods in the diagnosis of cancer

The usual methods for diagnosing and treating cancers are not very precise and work better for more advanced cases. Timely diagnosis of cancer is crucial because it increases the likelihood of successful treatment as with most of the diseases [2,3]. Using targeted therapies which focus on specific molecular and genetic issues can also help in reducing side effects [2,5].

Table 1 presents routine methods used for diagnosing cancers along with the accuracy, predictive value, costs and number of false positive/negatives [1–10].

### 2.1. Omics approaches in the diagnosis of cancer

Omics refers to a set of technologies and approaches used to study various aspects of biological systems on a large scale [15]. Various omics approaches, including genomics, epigenomics, transcriptomics, proteomics, and metabolomics, enable researchers to analyze molecules across different regulatory levels, with precision in both temporal and spatial dimensions. The integration of these approaches, known as ‘multi-omics’, uncovers insights not only from individual functional layers but also from the intricate interactions between them. This comprehensive view allows scientists to construct a multifaceted molecular framework, facilitating the analysis of information flow from root causes to functional outcomes [16].

In the context of cancer diagnosis, omics approaches involve the comprehensive analysis of various biological molecules to gain insights into the molecular and genetic basis of cancer. The approach to understanding complex disorders like cancer has evolved from a basic comparison of diseased and healthy cells in a slow and limited manner [15]. Omics methodologies have exerted a noteworthy influence in advancing the early diagnosis of cancer. These approaches include comprehensive analysis of biological molecules on a large scale to gain insights into the molecular profile of cancer cells. They also contribute to the identification of specific molecular signatures associated with cancer, enabling the development of more precise and personalized diagnostic tools for early detection. Integrating multiple omics data sets can provide a holistic understanding of the molecular landscape of cancer and enhance the accuracy of early diagnosis [17].

Table 2 highlights different omics approaches that provide comprehensive insights into cancer biology by analyzing various molecular components of cells. Each method has unique advantages, such as high sensitivity and non-invasive sample collection, but also presents challenges like complex data analysis, high costs, and the need for advanced technologies [15–24].

### 2.2. Metabolomics applications a promising technique for early diagnosis of cancer

Genetic and proteomic studies alone are insufficient to fully unravel the complexities of tissue cells, leaving a significant gap between genes and observable traits. Metabolomics, the study of small molecules in cells, tissues, and organisms, offers a direct link to molecular traits and complements other omics approaches [27]. This emerging field employs advanced analytical techniques such as nuclear magnetic resonance (NMR), mass spectrometry, and chromatography to characterize the metabolome, which includes both endogenous and exogenous metabolites. Compared to traditional clinical chemistry, metabolomics enables faster, more comprehensive, and cost-effective analysis, offering insights into the roles of metabolites in signaling, immune modulation, and environmental sensing. Metabolites are highly sensitive to internal and external factors, with even a single genetic variation capable of causing significant changes in metabolite levels, earning them the moniker “genomic canaries.” Despite over a million compounds comprising the human metabolome, only approximately 114,000 have been identified, underscoring the need for specialized workflows, including sample collection, metabolic quenching, extraction, and data interpretation [27,28].

Metabolomics is transforming cancer research by identifying disease-associated biomarkers and metabolic pathways, as well as monitoring treatment efficacy [17,29]. Cancer-related metabolic reprogramming reflects the altered cellular metabolism that sustains malignant proliferation and survival [30]. Exploring metabolomic signatures in diverse cancer types not only aids in detecting established malignancies but also reveals metabolic changes in precancerous lesions. This early detection is vital for preventive strategies and personalized therapies, offering a transformative approach to cancer management [17]. The advent of sophisticated analytics includes NMR spectroscopy and mass spectrometry (MS), coupled with advanced computational software, enables the simultaneous study and comparison of thousands of chemical compounds. The application of metabolomics has expanded small molecule biochemistry investigations, offering deeper insights into metabolic changes linked to diseases. When combined with other technologies like genomics and proteomics, it enhances diagnostic accuracy and helps identify personalized treatment strategies. This integrated approach is increasingly used in precision medicine, enabling earlier diagnosis and targeted therapies for complex diseases such as cancer and cardiovascular conditions. Understanding cancer cell metabolism could lead to safer and more effective treatments [31].

MS and NMR spectroscopy generate large datasets that can be challenging to interpret, and machine learning (ML) plays a critical role in enhancing their utility. ML algorithms excel at analyzing vast amounts of data, such as patient records, imaging, and test results, to identify subtle patterns and anomalies that might be overlooked by human observation [32]. This leads to more accurate diagnostics and facilitates early disease detection. In fields like radiology and pathology, ML models can analyze medical images such as X-rays, MRIs, and CT scans to detect anomalies like tumors or lesions with remarkable precision. ML enables personalized medicine by analyzing patient-specific data, including genetic predispositions, lifestyle factors, and medical histories [33]. This allows for the prediction of disease probability, onset, and progression, facilitating tailored treatment strategies and preventative measures. ML enables the combination of imaging data with omics information, such as genomics and proteomics, generating more reliable knowledge for diagnosis and treatment planning [32].

Combining dried serum spots (DSS) with nano-particle-enhanced laser desorption/ionization mass spectrometry (NPELDI MS) offers a groundbreaking approach to rapid, precise, and environmentally sustainable cancer diagnosis. This innovative method significantly reduces undiagnosed cases of colorectal, gastric, and pancreatic cancers, showcasing its potential for transformative and sustainable health gains [34]. DSS also minimizes the need for expensive, energy-intensive storage and transportation methods. This reduces waste and energy consumption, aligning with ecological considerations. The process requires only a minimal amount of blood (less than 0.05 mL), making it more accessible for widespread screening, even in low-resource settings. NPELDI MS offers distinct advantages over traditional mass spectrometry techniques. It eliminates time-consuming separation processes and reduces background noise in the low-mass range, improving the detection of cancer biomarkers. However the effectiveness of diagnostic tools can be influenced by the quality and type of biospecimens used, which may vary across populations and regions [35].

Nonetheless, the combination of DSS and NPELDI MS represents a significant step forward in sustainable metabolic diagnostics, offering maximum health gains with minimal environmental impact.

The pan-targeted quantification of the cancer serum proteome represents a significant leap forward in cancer diagnostics. By integrating various advanced proteomic strategies, researchers have developed comprehensive methodologies that enhance the sensitivity and specificity of cancer detection. This approach not only focuses on cancer-secreted proteins but also incorporates common serum proteins, creating a robust and multidimensional detection framework. Incorporating multiple techniques, such as data-independent acquisition proteomics, has significantly improved sensitivity and reproducibility in cancer diagnostics [36]. Multidimensional serum proteomic strategy achieved 87.5 % localization accuracy in multicancer diagnosis, covering breast, lung, stomach, liver, and colorectal cancers [36,37]. The Deep and Comprehensive Cancer Serum Protein Atlas constructed by Zhang and colleagues, includes over 2700 proteins, providing a valuable resource for identifying biomarkers and developing targeted mass spectrometry assays. It supports the development of proteomic-based assays and facilitates biomarker discovery. The use of standard peptide anchored parallel reaction monitoring enables precise quantification of cancer-related serum proteins with high throughput and sensitivity [37].

The method accelerates biomarker discovery and clinical translation by capturing a wide range of potential biomarkers across multiple cancer types. While the pan-targeted proteomic strategy shows great promise, challenges remain. Diverse populations and cancer types pose difficulties for standardizing methodologies, which may affect the generalizability of findings [37,38]. The combination of the Cancer Serum Atlas and advanced proteomic strategies offers a powerful tool for serological cancer studies. By providing high sensitivity, specificity, and comprehensive protein coverage, this approach has the potential to revolutionize multicancer detection, improving early diagnosis and patient outcomes [36].

Table 3 outlines the technological advancements in metabolomics that have revolutionized the timely diagnosis of cancer. The application of metabolomics has been pivotal in early detection, allowing for the identification of cancer-specific metabolic signatures at nascent stages. Furthermore, it plays a critical role in personalized medicine, enabling tailored treatment plans based on individual metabolic profiles. Integration with multi-omics approaches and machine learning applications has further refined precision oncology, improving diagnostic accuracy and treatment efficacy. Non-invasive diagnostic tools, such as metabolomic profiling of biofluids, have made cancer monitoring less invasive and more patient-friendly, while advancements in treatment monitoring ensure timely adjustments to therapeutic strategies [32,34,35,39–45].

## 3. Understanding the cancer metabolism: a primary approach for cancer diagnosis

In terms of treatment, it’s crucial to understand the mechanism at the cellular level. Therapeutic approaches for treating most conditions focus on impacting various energy-related processes, which includes glycolysis, the Krebs cycle, oxidative phosphorylation, glutamine use, breakdown of fatty acids, production of lipids, and the metabolism of amino acids [46–48].

Cancer cell metabolism is distinct from that of normal cells, characterized by altered metabolic pathways that support rapid growth and survival. One of the key features is the Warburg effect, where cancer cells rely heavily on glycolysis for energy production, even in the presence of oxygen, leading to lactate accumulation. This phenomenon is linked to mutations in oncogenes and tumor suppressor genes, such as MYC and TP53 [47,49]. This metabolic shift helps cancer cells generate biomass for proliferation and adapt to hypoxic tumor environments. Additionally, cancer cells often upregulate pathways involved in lipid and nucleotide synthesis, supporting cell membrane formation and DNA replication. These metabolic adaptations are not only essential for cancer progression but also offer potential therapeutic targets for disrupting tumor growth and survival [44].

### 3.1. Unveiling the metabolic divide: healthy vs. cancer cells

Healthy and cancer cells both require energy but diverge significantly in their metabolic strategies. Healthy cells efficiently utilize glucose, fats, and amino acids via oxidative phosphorylation, producing up to 32 ATP per glucose molecule. In contrast, cancer cells often favour aerobic glycolysis, converting glucose to lactate even in the presence of oxygen, yielding only 2 ATP per glucose molecule [46,49]. This inefficient process allows cancer cells to rapidly consume glucose and prioritize biomass production for growth. While healthy cells balance energy production with biosynthesis, cancer cells emphasize anabolic activity, consuming more amino acids and fatty acids to fuel uncontrolled proliferation and tumour formation [50]. Healthy cells maintain a balance resource used for energy production and biosynthesis at controlled rates. In contrast, cancer cells prioritize biosynthesis, consuming increased amounts of amino acids and fatty acids to support rapid proliferation and tumour growth, driving their aggressive behaviour [51].

Diversity in metabolic profiles across different cancer types and within specific tumour regions, underscores the necessity for personalized treatment approaches. Moreover, the adaptability of cancer cells to environmental changes and treatments emphasizes the importance of flexible therapeutic strategies [50,51]. Additionally, targeting the metabolic pathways of cancer cells presents promising opportunities for developing innovative treatments, thereby contributing to advancements in personalized [45,51,52].

Table 4 summarizes the distinctive metabolic characteristics of healthy cells versus cancer cells, focusing on their energy production, nutrient utilization, and regulation mechanisms. Healthy cells rely on oxidative phosphorylation and maintain balanced macromolecule synthesis for cellular homeostasis, while cancer cells exhibit reprogrammed metabolism driven by rapid proliferation demands. Key differences also include the Warburg effect, increased lactate production, and deregulated pathways in cancer cells that prioritize growth over efficiency [39,40,44–47,49,50,53].

## 4. Advanced metabolomics approaches for precise cancer diagnosis

### 4.1. Untargeted metabolomics approach

This method involves the global detection and measurement of all metabolites within a sample, followed by the analysis of metabolic networks and pathways to identify significant disruptions compared to controls. Untargeted metabolomics, also referred to as “unbiased” or “undirected” metabolomics, seeks to identify the maximum number of metabolites in a sample with the aim of classifying phenotypes based on observed metabolite configurations. This approach offers the potential to uncover novel biomarkers without prior knowledge [54,55].

### 4.2. Targeted metabolomics approach

In contrast, targeted metabolomics focuses on tracking and quantifying pre-identified metabolites associated with specific disease stages. This approach reduces the likelihood of false-positive results due to the predefined characteristics of the metabolites being analyzed. Targeted metabolomics, also termed “biased” or “directed” metabolomics, achieves greater precision by concentrating on a predetermined set of metabolites [55–57]. Studies of cancer predisposition genes in hematological malignancies and other cancers, including therapy-related myeloid neoplasms and malignancies without prior cytotoxic therapy, have revealed pathogenic germline variants such as CHEK2, BRCA1, DDX41, and TP53 as the most frequently identified. Notably, the frequency of these variants is higher in cancers without prior cytotoxic therapy compared to therapy-related myeloid neoplasms, with comparable rates observed between lymphoid and myeloid hematological malignancies [58].

### 4.3. Comparison of targeted and untargeted metabolomics approaches

The main drawback of targeted metabolomics lies in its restricted analysis of the metabolome, increasing the potential for missing the metabolomic of interest. On the other hand, unbiased or untargeted methods present the chance to unveil new biomarkers. Nonetheless, it’s important to note that this approach isn’t entirely impartial, as researchers select both the stationary phase and ionization mode, which can influence the detection of certain substances while potentially diminishing the detection of others [55].

Non-targeted metabolomics may also face challenges like misidentification of metabolites and bias or signal drift induced by matrix effects arise due to the absence of standards. Despite the existence of different signal-drift correction algorithms, their comparative effectiveness remains uncertain. Moreover, the lack of absolute quantification in non-targeted metabolomics impedes the determination of baseline metabolite levels and complicates result comparison between laboratories. In principle, the weaknesses inherent in one metabolomics approach are considered strengths in the other [55,57]. A study by Zhang et al. demonstrated that the targeted metabolomics approach for diagnosing lung cancer exhibited greater sensitivity, accuracy, and specificity compared to the non-targeted metabolomics approach. Ultimately, their findings indicated that both metabolomics strategies have the capacity to detect early-stage cancer and predict patient outcomes [57].

Integrating the advantages of both methodologies can provide a more comprehensive understanding of the metabolic alterations associated with early-stage cancer, thereby enhancing diagnostic capabilities and facilitating personalized treatment strategies [43]. Untargeted metabolomics is typically used for formulating hypotheses and biomarker discovery, while targeted methods are preferred for testing specific hypotheses or during validation. Despite its potential for biological insight, the utility of untargeted metabolomics is restricted by the challenge of identifying obscure metabolites [54].

**Fig. 1:**
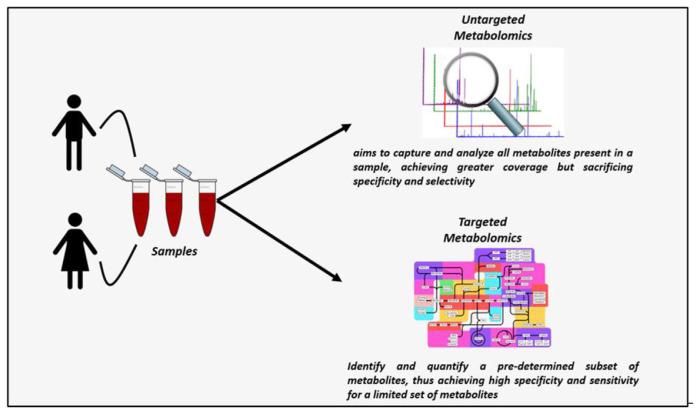
Comparison of targeted and untargeted metabolomics approach.

Fig. 1 displays the comparison of targeted and untargeted metabolomics approaches. The selection between targeted and untargeted metabolomics approaches for early cancer detection depends on the specific objectives of the study and the characteristics of the cancer being examined.

Table 5 highlights the differences between targeted and untargeted metabolomics approaches, focusing on their scope, sensitivity, and specificity. Targeted metabolomics is precise and highly sensitive, ideal for analyzing predefined metabolites, while untargeted metabolomics provides a broad and exploratory view of the entire metabolome, enabling novel biomarker discovery. Despite its complexity in data analysis and quantification, untargeted metabolomics offers a holistic perspective, complementing the specificity of targeted studies [31,55,57,59–61].

### 4.4. Metabolomic profiling procedure

To conduct a successful metabolomics study, careful planning of sample collection, storage, and preparation is crucial. While urine and blood are common samples, others like tissue and saliva are also used. For cancer studies, collecting samples at different times is important for accuracy [31,62,63]. During analysis both biofluid or tissue samples are examined from both healthy individuals and those with cancer. Advanced techniques like NMR [31,64] or mass spectrometry [61] are used to measure metabolites. The Metabolomics Standards Initiative provides guidelines for reporting standards, enhancing research credibility [52,60].

**Fig. 2:**
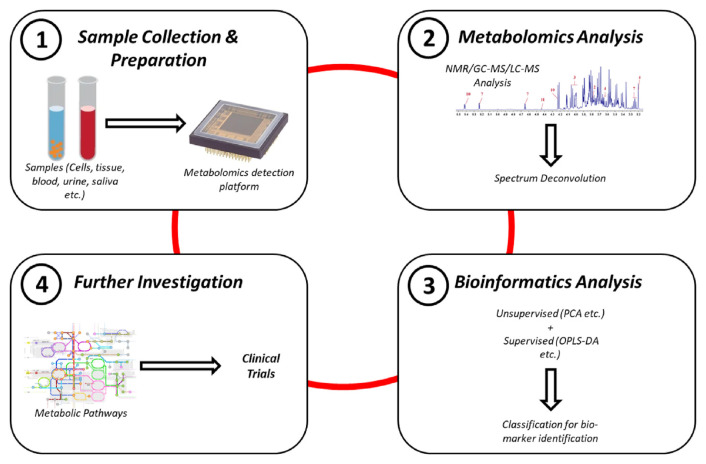
Diagrammatic representation of Metabolomics procedure in early Cancer diagnosis.

Fig. 2 illustrates the stepwise procedures in metabolomics that can aid in early cancer diagnosis. The diagram captures key stages, including sample collection, metabolite extraction, high-throughput analytical techniques (e.g., mass spectrometry or NMR spectroscopy), data processing, and biomarker identification. This streamlined approach facilitates the discovery of metabolic alterations associated with cancer, offering potential for early and non-invasive diagnosis.

#### 4.4.1. Important analytical techniques for metabolomic profiling

##### 4.4.1.1. NMR spectroscopy

NMR spectroscopy is used to provide a comprehensive profile of metabolites present in biological samples. Objective is to identify and quantify wide range of metabolites, enabling detection of metabolic alterations associated with the conditions under study. Biological samples (e.g., blood, urine, tissue extracts) are collected and prepared following standardized protocols. Samples are homogenized, and metabolites are extracted using appropriate solvents, ensuring consistency and reproducibility. The extracts were then lyophilized and reconstituted in deuterated solvents for NMR analysis [65].

The analytical procedure encompasses several critical steps to ensure accurate metabolite characterization. Initially, the NMR spectrometer is calibrated using a standard reference compound. Subsequently, samples are analyzed on a high-field NMR spectrometer (e.g., 600MHz), where both one-dimensional) and two-dimensional NMR spectra, including COSY and HSQC experiments, are acquired to provide comprehensive structural information. The obtained spectral data are processed with software tools such as TopSpin and Chenomx, incorporating essential procedures like baseline correction, phase adjustment, and peak alignment to minimize artifacts and ensure precise interpretation [31,65] Metabolites are then identified by comparing their chemical shifts, coupling constants, and peak patterns with established spectral databases and relevant literature. Quantification is performed using the area under the curve of specific peaks, normalized against an internal standard to enhance the accuracy and reliability of metabolite concentration assessments. To ensure data reliability, quality control samples are included in each batch, consisting of pooled biological samples representative of the study population. Additionally, replicate analyses and consistency checks are conducted to validate the reproducibility of the NMR measurements [60,66].

##### 4.4.1.2. Mass spectrometry (MS)

MS was used to complement NMR data by providing high sensitivity and specificity in detecting and quantifying metabolites, particularly those present at low concentrations. MS enables the detailed analysis of the metabolic profile, including the identification of unknown compounds and the elucidation of complex metabolic pathways [67].

Sample preparation involves extraction and purification steps, similar to those used in NMR analysis. For MS, samples are often derivatized to enhance their volatility and ionization efficiency, particularly in gas chromatography-mass spectrometry applications. The analytical procedure begins with instrument calibration, where the mass spectrometer is calibrated using a set of standard compounds with known mass-to-charge ratios (m/z). Metabolites are then separated using either gas chromatography or liquid chromatography before MS analysis, with gas chromatography-mass spectrometry (GC–MS) or liquid chromatography-mass spectrometry (LC–MS) selected based on the physicochemical properties of the target metabolites. Ionization techniques, such as electron ionization for GC–MS or electrospray ionization for LC–MS, are employed to generate ions from the sample molecules. The MS analyzes these ions, recording their m/z ratios [68].

For MS/MS analysis, precursor ions are fragmented, and the resulting product ions are examined to provide structural information. The mass spectra are processed using software tools like Xcalibur and MetaboAnalyst, where peak detection, deconvolution, and alignment are performed to generate accurate mass profiles. Metabolites are identified by comparing MS spectra with reference databases (e.g., METLIN, HMDB), and quantification is achieved using calibration curves constructed from standard compounds, with internal standards employed for normalization. Quality control measures include the use of blank samples, spiked samples, and replicate analyses to monitor and correct for analytical variability, while isotopic standards are used to ensure accuracy in quantification [52,66].

Data from NMR and MS can be integrated to provide a comprehensive metabolomic profile. Multivariate statistical analysis, including principal component analysis and partial least squares-discriminant analysis, are used to interpret the data and identify significant metabolic changes [52,64].

## 5. Application of metabolomics in cancer detection: current practices

Recent advancements in non-invasive testing have integrated untargeted serum metabolomics with machine learning algorithms, achieving over 99 % accuracy in detecting multiple cancers at early stages, while also identifying the tissue of origin [66,69]. Liquid biopsy applications, utilizing circulating metabolites as real-time indicators, have demonstrated significant promise in cancer detection, with blood-based NMR metabolomics achieving a 95 % detection rate. Metabolomics also enables the monitoring of dynamic changes within tumor microenvironments, allowing for the identification of early biomarkers and assessment of treatment responses through the analysis of biofluids such as blood or urine [65,66,70].

**Fig. 3:**
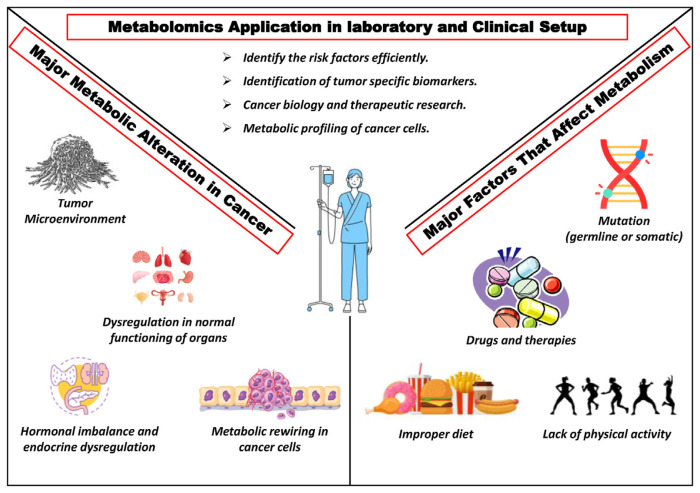
Diagrammatic representation of metabolomics applications in early Cancer detection.

Fig. 3 depicts critical aspects such as the tumor microenvironment, metabolic rewiring in cancer cells, hormonal imbalances, and the role of mutations, as well as external factors like improper diet, lack of physical activity, and drug interactions. These interconnected elements emphasize the comprehensive approach metabolomics offers for understanding cancer biology and identifying early diagnostic biomarkers.

### 5.1. Metabolic signatures

Metabolomic profiling is pivotal in identifying specific metabolites or patterns associated with early cancer stages. These metabolites include a diverse array of compounds, such as peptides, sugars, and organic acids, which reflect genetic, proteomic, and environmental influences [71–73]. Untargeted metabolomics enables the analysis of “metabolic risk factors,” which can indicate an increased likelihood of developing certain cancers [35,74]. For example, dietary fat intake has been associated with colorectal cancer risk, while fatty acid levels have been linked to prostate cancer. Alterations in metabolites involved in lipid, amino acid, and glucose metabolism have been connected to prostate cancer susceptibility. Analysis of pre-diagnostic serum in prostate cancer cases revealed a direct correlation between omega-6 polyunsaturated fatty acids and increased cancer risk, whereas omega-3 polyunsaturated fatty acids demonstrated an inverse relationship [50,62,75]. Furthermore, specific polyunsaturated fatty acids have also been implicated as risk factors for breast cancer [76].

In aggressive lung cancers elevated lactate production is seen, indicating that lactate levels may serve as a marker for disease severity [62,77]. High level of glutamate levels were also observed in mammary carcinoma tissue, whereas, colon and stomach tumor tissue exhibit reduced levels of glutamate due to heightened rates of glutaminolysis, which in turn induced cell proliferation [76,78,79]. Additional metabolomic investigations utilizing pre-cancer diagnosis serum in screening or preventive studies have established correlations between branched-chain amino acids & pancreatic cancer, as well as between pseudo uridine and ovarian cancer as reviewed by Schmidt et al. [43]. As many as 5661 small metabolites have been identified in urine [80–82], whereas in blood as many as 38,036 metabolites were identified [61,62]. The ongoing discovery of numerous small metabolites over recent decades highlights their bioactivity and elucidates the possibility of biological significance in disease progression [71].

#### 5.1.1. Metabolic fingerprinting in diagnosis

The study by Xu et al. highlights the potential of plasma metabolic fingerprinting (PMF) as a novel diagnostic and prognostic tool for gastric cancer (GC). This large-scale, multicenter research aimed to develop diagnostic and prognostic models for GC based on PMFs, leveraging unique metabolic profiles associated with the disease to enable early detection and improved patient outcomes. Using nanoparticle-enhanced laser desorption/ionization-mass spectrometry, the researchers obtained PMFs, demonstrating high throughput and reproducibility. In addition to its diagnostic applications, the study developed prognostic models based on PMFs using Cox regression, revealing distinct metabolic subtypes of GC that correlate with overall survival rates and specific metabolic pathways, such as the tricarboxylic acid cycle and lipid metabolism. The identification of metabolic biomarkers, including N-formylkynurenine and creatine riboside, offers new avenues for prognosis and targeted therapies [83]. Despite these promising results, challenges remain. The heterogeneity of GC and the complexity of metabolic interactions pose obstacles to clinical application. Furthermore, traditional histopathological methods continue to serve as the gold standard for GC diagnosis, underscoring the need for a balanced approach in clinical practice [83,84].

### 5.2. Biomarkers

Metabolomics plays a pivotal role in uncovering potential biomarkers, acting as dependable indicators for the early detection of cancer. These biomarkers are evident across a range of biological samples, spanning blood, urine, tissue, and even analysis of exhaled breath. Notably, volatile organic compounds (VOCs) found in breath and breath condensate have emerged as valuable biomarkers, particularly for the detection of lung cancer [85].

#### 5.2.1. Blood biomarkers

The integration of metabolomics has provided notable advancements in identifying blood biomarkers across diverse tumors, such as pancreatic [86], liver [87], lung [85], and breast cancers [88]. Zhang et al. identified and validated plasma metabolite biomarkers for early-stage non-small cell lung cancer (NSCLC) diagnosis using targeted mass spectrometry on plasma samples. Key metabolites, including β-hydroxybutyric acid, LysoPC 20:3, PC ae C40:6, citric acid, and fumaric acid, showed significant differences between healthy individuals and stage I/II NSCLC patients. Predictive models based on these biomarkers demonstrated excellent diagnostic performance. This study highlights the potential of metabolite-based tests for the early detection of NSCLC [89]. NMR spectroscopy has been used to investigate metabolic alterations in thyroid tissues and plasma from patients with papillary thyroid microcarcinoma and reduced fatty acid levels and heightened concentrations of various amino acids in thyroid tissues. This underlines the potential of metabolomics to offer sensitive diagnostic outcomes and comprehensive therapeutic insights across various tumour types [42]. In pancreatic cancer, researchers identified five biomarkers creatine, inosine, beta-sitosterol, sphinganine and glycocholic acid using a metabolomics approach with potential clinical utility for diagnosis [90,91]. Mayerle et al. demonstrated the identification of biomarker signatures for differentiating pancreatic ductal adenocarcinoma and chronic pancreatitis using untargeted metabolomics techniques [86].

#### 5.2.2. Urine biomarkers

Urine biomarkers represent a non-invasive and practical tool for disease screening, particularly in asymptomatic individuals and populations at elevated risk [92]. By enabling direct comparisons between patients and healthy controls, urine-based assays facilitate early detection and differentiation of pathological conditions, making them suitable for broader screening applications. This approach improves the likelihood of identifying cancers at earlier, more treatable stages [55,82]. Additionally, urine biomarkers provide a convenient method for monitoring disease progression and therapeutic response, supporting personalized and timely clinical interventions. Accurate analysis of urine metabolites has the potential to enhance understanding of tumor pathology and advance clinical practices [63,80].

In urological cancers, elevated levels of specific metabolites, such as acylcarnitine, have been associated with cancer status and used to grade renal cancer [93]. Similarly, abnormalities in fatty acid metabolism in bladder cancer have been linked to increased levels of acetylcarnitine and adipate in urine, indicative of tumorigenesis [94,95]. Furthermore, differential urinary metabolites have been identified in various cancers, including liver [96], stomach [97], cervical [98], ovarian, and breast cancers [99]. For example, elevated levels of 1-methyl adenosine, 1-methylguanosine, and 8-hydroxy-2′-deoxyguanosine in urine have been associated with early-stage breast cancer. These findings highlight the diagnostic potential of urine biomarkers in providing unique insights into specific cancer types, ultimately improving early detection and patient outcomes [81,100].

#### 5.2.3. Salivary biomarkers

Other biological fluids hold promise for early cancer detection. Among these, saliva—composed predominantly of water, along with minor amounts of proteins, electrolytes, and low-molecular-weight components—plays a crucial role in oral health and digestion. Recent advancements in analytical techniques, such as capillary electrophoresis time-of-flight mass spectrometry, have identified cancer-specific metabolites in saliva [101]. Notably, elevated concentrations of certain metabolites, including polyamines, taurine, and piperidine, have been linked to oral cancer. The integration of saliva metabolomics with tumor metabolomics offers a promising avenue for non-invasive cancer screening. Moreover, ongoing research into salivary biomarkers across a spectrum of cancers and other diseases signifies considerable progress toward their potential clinical applications in cancer detection [102].

#### 5.2.4. Cerebrospinal fluid biomarkers

Cerebrospinal fluid (CSF) analysis provides valuable insights into neurological and biochemical alterations, particularly in cases of central nervous system dysfunction. Recent studies have identified specific metabolites in CSF that are linked to glioma, highlighting the potential of mass spectrometry techniques in biomarker identification and disease classification [103–105]. Recent research using untargeted metabolomics has identified metabolic differences in CSF samples from various brain tumors, showing promise for brain tumor diagnosis [72,106,107]. Metabolites like tricarboxylic acid cycle products, tryptophan, and methionine have been detected in cerebrospinal fluid of gliomas and metastases [108,109]. Inflammatory markers in CSF, such as interleukin-10 and soluble interleukin-receptor, may aid in CNS tumor diagnosis, particularly in primary CNS lymphoma [110,111]. Specific CSF biomarkers could potentially guide surgical planning, minimizing the need for high-risk procedures like biopsy and craniotomy [100–112].

#### 5.2.5. VOC as biomarkers

In addition to tissue and biofluids, researchers have investigated exhaled breath as a promising source of neoplasm biomarkers [113]. VOC detected during breath exhalation has shown promise in distinguishing patients with non-small cell lung cancer from non-cancer controls [87,114–116]. In lung cancer, key biomarkers identified include 2-butanone, 1-propanol, along with isoprene, ethylbenzene, styrene, and hexanal, as highlighted in a review by Saalberg et al. [85]. More recently, Wang et al. identified 16 VOCs associated with lung cancer, including aldehydes, ketones, hydrocarbons, carboxylic acids, and furans [117]. These VOCs serve as potential biomarkers for early detection, reflecting the metabolic alterations characteristic of lung cancer development. In breast cancer, VOC biomarkers include phosphocholine, isoleucine, threonine, glutamate, histidine, acetoacetate, glycerol, mannose, phenylalanine, and pyruvate [88,118–120].

**Fig. 4:**
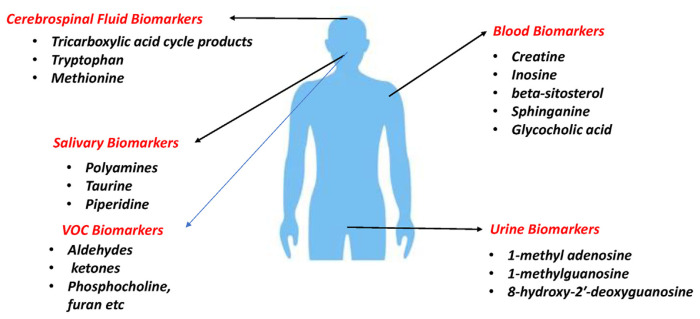
Diagrammatic representation of several biomarkers for cancer.

Fig. 4 illustrates various biomarkers with potential applications in cancer diagnosis. It includes cerebrospinal fluid biomarkers like tricarboxylic acid cycle products, tryptophan, and methionine, as well as blood biomarkers such as creatine, inosine, and glycocholic acid. Additionally, salivary, urinary, and VOC biomarkers provide a non-invasive approach, broadening the scope for early cancer detection.

### 5.3. Non-invasive detection

A key advantage of metabolomics lies in its capacity to enable non-invasive cancer detection methods. This is particularly demonstrated in the analysis of readily accessible biological fluids, such as blood and urine [27,115]. Moreover, the emerging field of breath analysis for VOCs presents promising potential as an additional non-invasive approach for cancer detection [69,116,121]. Positron-emission tomography is an imaging technique that relies on the heightened glucose uptake of tumors relative to normal cells. It utilizes a radiolabeled glucose analogue, 2-[18F] fluoro-2-deoxy-d-glucose, to identify tumor cell glucose uptake [122]. Magnetic Resonance Spectroscopic Imaging (MRSI), which acquires an NMR spectrum from a small 3D tissue sample, offers advantages over standard MRI by detecting multiple metabolic modifications in tumors beyond glucose uptake [123,124]. Choline detection through MRSI has proven valuable in diagnosing breast, brain and prostate cancer [125,126]. A study by Guo et al. using high-resolution label-free molecular imaging for brain tumors marks a groundbreaking advancement in medical diagnostics. This innovative technique enhances tumor visualization without the need for contrast agents, offering significant potential for improving patient outcomes. The technique delivers detailed visualization of tumor morphology, facilitating more precise diagnosis and treatment planning with the identification of brain tumour metabolites such as choline, lactate and N-acetyl aspartate, by integrating with machine learning [127]. Remarkably, MRSI’s choline detection exhibits 100 % sensitivity in distinguishing malignant from benign breast tumors, potentially reducing unnecessary breast biopsies [118,125,126]. While tissue biopsy remains essential for the initial diagnosis, it is neither feasible nor preferable for cancer screening, monitoring, or surveillance. Conversely, biofluids like blood, serum, urine, saliva, and sweat offer a convenient source for biomarker detection [127].

### 5.4. Modern metabolomic tools for diagnosis of cancer metastasis

Quantitative metabolomics is an invaluable analytical resource for examining metabolites in biological samples. Established techniques such as NMR spectroscopy, GC–MS and LC–MS, developed decades ago, have played significant roles in identifying and quantifying metabolites in cancer metastasis [32,128,129]. Nevertheless, these methods necessitate a relatively large quantity of cancer cells (>10,000 cells) and are unable to differentiate metabolic differences at the individual cellular level. Recognizing the metabolic variability within a neoplasm is essential as variations among specific groups of cancer cells can impact metastasis efficiency and treatment response. Pinpointing distinct metabolic characteristics in various subsets of cancer cells could provide predictive clues regarding which cells are prone to metastasize effectively, potentially serving as diagnostic and indicative biomarkers [130].

Computer-based platforms are vital for analyzing data in metabolomic studies, utilizing databases, networks, and tools. Over time, these tools have evolved to handle vast datasets [130,131]. While well-annotated chromatography methods can identify known metabolites, LC–MS and GC–MS techniques often leave many peaks unannotated, requiring further analysis. Tools such as mzMINE, XCMS, and Global Natural Products Social Networking (GNPS) have enhanced data processing [131,132]. GNPS acts as a repository for raw MS data, aiding in metabolite identification. Although not yet widely implemented in tumor studies, Flux Balance Analysis provides valuable insights into the metabolic functioning of tumor cells [133]. MetaboAnalyst facilitates data normalization, statistical and functional analysis, widely used in metabolic studies. Recent advancements like the NetID algorithm and LD-Portal have emerged for metabolite discovery [134]. Chong et al. introduced MetaboAnalyst 4.0, a significant upgrade to the MetaboAnalyst platform, in Nucleic Acids Research. This version features several key advancements aimed at enhancing transparency and integrative capabilities in metabolomics analysis. The MS Peaks to Pathways module was added, enabling the prediction of pathway activity from untargeted mass spectral data, while the Biomarker Meta-analysis module facilitates robust biomarker identification through the integration of multiple metabolomic datasets. Furthermore, foundational knowledge bases including compound libraries, metabolite sets, and metabolic pathways were updated using the latest data from the Human Metabolome Database (HMDB), ensuring accurate and up-to-date analysis. Together, these enhancements establish MetaboAnalyst 4.0 as a robust and versatile platform for metabolomics research [135]. The HMDB serves as an ideal database for cancer metabolomics, providing rich chemical information and enhancing compound identification in MetaboAnalyst [134].

### 5.5. Early detection across cancer types

The application of metabolomics is not limited to a particular cancer type. It has been successfully employed across various cancers, including breast, colorectal, prostate, as per recent systematic review study by Navarro Ledesma S. et al. [136].

#### 5.5.1. Breast cancer

Polyamines, including spermine and spermidine, are implicated as potential oncometabolites in breast cancer, associated with accelerated tumor growth through their involvement in cellular biogenesis and accumulation in various tissues. Evidence indicates that elevated concentrations of polyamines are present in the plasma and urine of breast cancer patients, suggesting their role in disease progression. The dysregulation of polyamine metabolism may contribute to the malignant phenotype by promoting cell proliferation, inhibiting apoptosis, and facilitating tumor microenvironment changes, thereby highlighting their potential as biomarkers for breast cancer diagnosis and prognosis [74,136]. Breast cancer patients display significant alterations in choline metabolism associated with estrogen, alongside increased levels of metabolites linked to oxidative DNA damage and methylation processes. The cytidine-5-phosphate/pentadecanoic acid ratio was assessed for its accuracy and precision as a potential biomarker in breast cancer, demonstrating notable efficacy as a key indicator of the disease. This biomarker exhibited a sensitivity of 94.8 % and specificity of 93.9 %, highlighting its potential utility in clinical diagnostics [137]. Research studies identified taurine, hypotaurine, glutamate, and aspartate pathways as crucial biomarkers for timely breast cancer diagnosis using LC–MS and GC-MS techniques [76,99,120,137]. Vaida et al. introduced a novel approach for early breast cancer detection using metabolomic biomarkers and machine learning. They identified 11-biomarker panel including 9 metabolites, a metabolite ratio (kynurenine-to-tryptophan), and the demographic variable age. Machine learning techniques, including Naive Bayes, Support Vector Classifier, and Principal Component Analysis, are combined to enhance biomarker selection. The study demonstrates the potential of metabolomics to improve early detection and provide a more personalized, non-invasive screening method, offering a promising alternative to traditional approaches like mammography [41].

#### 5.5.2. Prostate cancer

Oncometabolites such as choline, glucose, nitric oxide and uric acid are implicated in prostate cancer. While these metabolites are linked to disease severity and mortality, interventions targeting uric acid reduction have not significantly impacted tumor growth or overall survival [67]. Prostate-specific antigen (PSA) is commonly used for prostate cancer (PCa) screening but has low sensitivity and specificity, leading to false positives, unnecessary biopsies, and missed significant cases. Novel biomarkers like the Prostate Health Index (FDA-approved in 2012) and the 4K Score improve diagnostic accuracy and reduce unnecessary biopsies by 40 % and 43 %, respectively, while maintaining sensitivity for aggressive PCa. PCA3, an FDA-approved urine test, aids in repeat biopsy decisions, though it is less effective for predicting aggressive cancer. Combining PCA3 with TMPRSS2:ERG gene fusion enhances diagnostic precision. Non-invasive tests like ExoDx Prostate IntelliScore and SelectMDx show promise in predicting high-grade PCa and minimizing biopsies. Despite these advancements, further validation is required, and PSA remains the primary biomarker for PCa detection [138].

#### 5.5.3. Colon cancer

Colon cancer has been associated with specific metabolite including methionine which plays a key role in tumour growth and metabolism. Recent studies show that methionine depletion through dietary intervention shows promise in managing colon cancer [139]. Kynurenine is also identified as an oncometabolite in colon cancer [140]. Studies underscore the role of diet and lifestyle in colorectal cancer development, with high methionine intake from red meat serving as a significant risk factor [139]. Additionally, colon cancer progression appears to be related to other oncometabolites within the acyl-CoA synthetase/stearoyl-CoA desaturase lipid network [141,142]. Increased levels of 3′-methoxyguanosine, histamine, phenyllactic acid, citraconic acid, guanosine, cholesterol sulfate, β-d-glucosamine, L-2-aminoadipic acid was observed in cervical cancer tissues, also, a study by Jia et al. suggested a close relation between taurine and its metabolic pathways with cervical cancer [98]. Several biomarkers were identified as being associated with abnormal fatty acid oxidation and neutrophil functions, as well as phospholipid and bile acid metabolism in ovarian cancer and other diseases [45,48,99,143].

## 6. Application of metabolomics in cancer therapeutic strategies

In clinical settings, metabolomics has emerged as a valuable tool for identifying metabolites that reflect the effects of anticancer medications, thereby improving drug efficacy and reducing unnecessary adverse reactions [144]. Han et al. highlight the diverse applications of metabolomics in cancer treatment [100]. For instance, the chemotherapeutic agent Adriamycin has been shown to interact with specific metabolite levels in cancer patients, providing insights into its mechanism of action [145]. Historically, the earliest medical interventions for cancer employed “antimetabolites,” compounds that disrupt metabolic pathways by mimicking endogenous metabolites [80,146]. Antimetabolites, including cytarabine, gemcitabine, 5-fluorouracil (5-FU), and methotrexate, primarily target DNA synthesis in advanced-stage cancer [91,145,147,148]. Recent research has explored molecular features influencing responses to antimetabolite chemotherapies, emphasizing the critical role of metabolic pathways in determining treatment outcomes. A novel computational method identified gene expression signatures associated with sensitivity to various antimetabolite agents, revealing distinct metabolic expression patterns for drugs like 5-FU and gemcitabine. These findings underscore the importance of metabolic context in driving tailored therapeutic responses [91].

Lipidomics, a specialized branch of metabolomics, focuses on the comprehensive analysis of lipid species within biological systems. This subfield has garnered significant attention in the context of anticancer treatments, as lipids play crucial roles in cell signalling, membrane structure, and energy storage, all of which are pivotal in cancer biology [149]. Studies utilizing lipidomic approaches have been conducted using both patient samples and in vitro models to elucidate the alterations in lipid profiles associated with various cancer types and treatment responses [149,150]. By examining the changes in lipid metabolism during chemotherapy or targeted therapies, researchers aim to identify potential lipid biomarkers that could serve as indicators of treatment efficacy, resistance, or disease progression [23,151]. Additionally, lipidomic analyses can provide insights into the mechanisms underlying cancer cell survival and proliferation, highlighting how specific lipid species contribute to tumor growth and metastasis [150]. Ultimately, lipidomics offers promising avenues for developing novel therapeutic strategies and enhancing personalized medicine in cancer treatment [23,151,152]. Among the extensively studied drugs, metformin, originally developed for the treatment of type II diabetes, has garnered attention for its potential anticancer properties. This drug exerts its effects primarily through the inhibition of mitochondrial complex I, leading to a reduction in ATP production and subsequent energy stress within cancer cells. The induced energy deficit can trigger various metabolic adaptations in tumor cells, promoting cell cycle arrest and apoptosis. Additionally, metformin has been shown to activate the AMP-activated protein kinase pathway, which further contributes to its antitumor effects by inhibiting anabolic processes and enhancing catabolic pathways. These mechanisms collectively underscore metformin’s potential as a therapeutic agent in oncology, highlighting its role in targeting metabolic vulnerabilities in cancer cells [153,154].

Giordano et al. highlighted the potential of curcumin as a long-term anticancer agent, demonstrating its effectiveness in inhibiting tumor growth and promoting apoptosis in breast cancer cells [155]. Furthermore, selenium IV injections have been explored for their role in alleviating symptoms associated with breast cancer-related lymphedema, offering a therapeutic approach to enhance patient quality of life [156]. These findings emphasize the importance of dietary and supplemental interventions in the management of breast cancer and its related complications. In prostate cancer, neoadjuvant therapy significantly suppressed key pathways involved in biosynthesis and energy metabolism within tumor tissue. This inhibition holds promise for preventing tumor growth and metastasis [157].

### 6.1. Role of metabolomics for assessing drug resistance in cancer

The metabolic behaviour of cancer cells undergoes significant alterations when they develop resistance to therapeutic agents. Notably, the target drug can induce distinct metabolic changes in drug-sensitive cells compared to those that have acquired resistance. This phenomenon underscores the utility of metabolomics in detecting these metabolic shifts and understanding cellular responses to treatment. By identifying the metabolic profiles associated with drug intractability, metabolomics facilitates early monitoring of drug resistance. This rapid and efficient analytical approach assesses metabolic outcomes across various physiological and pathological states, enabling the prediction and evaluation of patients’ sensitivity to chemotherapy and their likelihood of developing drug resistance [158]. Furthermore, analysing the metabolic patterns of individual cancer patients can reveal differences between drug-resistant and drug-sensitive phenotypes, thereby aiding in the early detection of resistance and informing timely treatment modifications. Overall, metabolomics has significantly advanced our understanding of the genetic underpinnings of drug resistance in tumors [51,158].

### 6.2. Challenges in metabolomic application for early cancer detection and treatments

Metabolomics holds immense promise for early cancer detection, but significant challenges must be overcome to realize its clinical utility. Addressing issues related to sample collection, analytical techniques, data analysis, validation, and clinical translation is essential for advancing metabolomic approaches in the context of early cancer detection. Collaborative efforts among researchers, clinicians, and industry stakeholders are crucial for overcoming these challenges and harnessing the full potential of metabolomics in improving cancer diagnosis and patient outcomes [44,45]. While most metabolic drugs don’t differentiate between cancer and normal cells, some have been designed to specifically target mutated enzymes found in cancer cells. This approach has shown promise, particularly in treating acute myeloid leukemia with isocitrate dehydrogenase (IDH) mutations. IDH inhibitors are also being tested in clinical trials for other IDH-mutated cancers like glioma, chondrosarcoma, and intrahepatic cholangiocarcinoma [159–161]. However, early research suggests that these inhibitors may be less effective in solid tumors. This could be because mutant IDH activity might primarily drive cancer initiation by changing certain genetic factors but might be less critical in maintaining tumors as they progress [162]. It’s important to note that while drugs targeting metabolic enzymes can slow down tumor growth in lab studies, they rarely cause tumors to shrink completely. This suggests that these drugs might work best when combined with other treatments or used to maintain progress after initial therapy. The primary obstacles in metabolomics involve handling the extensive volumes of metabolic data and navigating the intricate chemical compositions of metabolites [163].

## 7. Discussion

Metabolomics holds immense potential for revolutionizing cancer diagnosis and treatment. Its ability to detect early metabolic changes and provide personalized insights into disease progression and therapy response makes it a valuable tool in the clinical setting [130,164–166]. Continued advancements in analytical techniques and data analysis methods will further enhance the clinical applications of metabolomics, ultimately improving patient outcomes and advancing the field of personalized medicine [21,44,167]. Additionally, metabolomic profiling can monitor treatment response and disease progression in real-time. By comparing pre- and post-treatment metabolomic profiles, clinicians can assess the efficacy of the therapy and make informed decisions about continuing, adjusting, or switching treatments. For example, changes in metabolite levels can indicate tumor regression or resistance to chemotherapy, allowing for timely modifications to the treatment regimen. Metabolomics can uncover novel therapeutic targets by elucidating the metabolic pathways dysregulated in cancer. Targeting these pathways with specific drugs can improve treatment efficacy and reduce side effects. For instance, inhibitors of key metabolic enzymes identified through metabolomic studies have shown promise in preclinical models and early-phase clinical trials. The integration of metabolomics with other ‘omics’ technologies, such as genomics, proteomics, and transcriptomics, is driving significant advancements in biology and medicine. These integrated approaches enhance our understanding of disease mechanisms and facilitate the identification of early biomarkers for various diseases, contributing to more accurate and earlier diagnosis [27]. Moreover, the comprehensive data provided by these ‘omics’ technologies is invaluable for bioinformatics, enabling the development of sophisticated algorithms and models that can predict disease outcomes and responses to therapies [164,168]. This holistic view of biological systems is not only transforming drug discovery processes by identifying novel therapeutic targets but also paving the way for the development of more effective and personalized treatment strategies. By leveraging the strengths of multiple ‘omics’ disciplines, we are witnessing a paradigm shift in disease diagnosis, treatment, and prevention, underscoring the pivotal role of these advanced biological methods in modern healthcare [159,160]. Early detection of cancer significantly improves treatment outcomes and survival rates. Traditional diagnostic methods, such as imaging and histopathology, often detect cancer at advanced stages when therapeutic options are limited. Metabolomics presents promising alternative due to its ability to detect subtle metabolic alterations associated with tumorigenesis at an early stage. Cancer cells exhibit distinct metabolic reprogramming to support their rapid growth and proliferation. Metabolomic profiling can identify specific metabolic biomarkers indicative of early-stage cancer. For instance, altered levels of amino acids, lipids, and glycolytic intermediates have been observed in various cancers. The identification of these biomarkers can lead to development of noninvasive diagnostic tests, such as blood or urine tests, which can be used for early cancer screening. The sensitivity and specificity of metabolomic approaches are crucial for early cancer detection [169–171]. Advanced analytical techniques, including NMR spectroscopy and MS, enable the detection of minute changes in metabolite concentrations. These technologies, coupled with sophisticated data analysis methods, can discern cancer-specific metabolic signatures from those of benign conditions, reducing false-positive rates and enhancing diagnostic accuracy. Several studies have demonstrated potential of metabolomics in early cancer detection. For example, in breast cancer, specific metabolite patterns in serum have been correlated with early disease stages. Similarly, urinary metabolomic profiles have been used to detect prostate cancer with high sensitivity. Ongoing clinical trials are further validating these findings and paving the way for the integration of metabolomic biomarkers into routine clinical practice [171,172].

Despite the immense potential of metabolomics in cancer diagnosis and treatment, several challenges and limitations must be addressed to optimize its clinical utility. One significant limitation is the lack of standardized protocols across metabolomics studies making it difficult to compare findings across studies or to replicate them reliably in independent settings. Moreover, the sensitivity of metabolomic profiling depends heavily on the analytical techniques employed, and current technologies may still miss low-abundance metabolites, particularly in heterogeneous tumor samples where concentrations of relevant biomarkers can vary significantly. Another challenge lies in the complexity of data interpretation. While advances in bioinformatics have facilitated this process, the integration of metabolomic data with other ‘omics’ technologies, such as genomics and proteomics, is still in its early stages, and further development is needed to fully leverage the power of these combined datasets. Additionally, while metabolomic profiling has shown promise in early cancer detection and personalized treatment, the identification of specific metabolic biomarkers that are universally applicable across different patient populations remains difficult. Finally, translating metabolomic discoveries into routine clinical practice faces regulatory and validation hurdles. The cost and complexity of current metabolomic techniques, as well as the need for extensive validation in diverse clinical settings, pose barriers to widespread use [173,174].

In the context of clinical applications, metabolomics plays a crucial role by detecting subtle metabolic changes at the molecular level, enabling the identification of early-stage biomarkers, often before clinical symptoms manifest. This non-invasive approach, utilizing biofluids such as blood, urine, or saliva, enhances patient compliance and minimizes the risks associated with invasive diagnostic procedures such as biopsies. Furthermore, metabolomics-based tests are particularly valuable in monitoring high-risk populations, enabling timely interventions and reducing cancer-related morbidity and mortality. The integration of metabolomics into clinical workflows is increasingly facilitated by advancements in analytical technologies such as NMR spectroscopy and MS. These techniques provide high sensitivity and specificity in detecting cancer-related metabolic alterations, ensuring reliable diagnostic outputs [175]. Additionally, computational tools, including machine learning algorithms, are now being employed to analyze complex metabolomic datasets, improving diagnostic accuracy and predictive power. Metabolomics-based tests have demonstrated the ability to distinguish between benign and malignant lesions, stratify patients by disease stage, and even predict treatment responses, further establishing their clinical relevance. A critical factor influencing the clinical acceptability of metabolomics is its compatibility with existing diagnostic protocols. By complementing traditional imaging and genomic methods, metabolomics provides a multi-dimensional diagnostic perspective, enhancing the overall accuracy of early cancer detection [176,177].

Several studies, including those reviewed herein, have validated the use of metabolomic biomarkers in clinical settings for cancers such as breast, colorectal, and lung cancer. Ongoing clinical trials are further exploring the utility of metabolomics-based liquid biopsies, which could offer a cost-effective alternative to tissue-based diagnostics. While the clinical adoption of metabolomics is promising, challenges remain, including the need for standardized protocols, large-scale validation of biomarkers, and regulatory approvals. Addressing these challenges through collaborative efforts between researchers, clinicians, and regulatory bodies is crucial for translating metabolomic insights into routine clinical practice. With continued advancements, metabolomics holds immense potential to revolutionize cancer diagnostics by enabling earlier detection, personalized treatment strategies, and improved patient outcomes.

## 8. Conclusion

This review provides a comprehensive analysis of metabolomics as a promising tool for early cancer detection, with a particular focus on its integration with advanced analytical techniques such as NMR spectroscopy and MS. Unlike prior reviews, which often examine isolated aspects of metabolomics or focus on single cancer types, this work explores the broader potential of metabolomics in identifying early-stage biomarkers across diverse cancers. The novelty of this review lies in its critical evaluation of metabolomics in the context of emerging technologies and its ability to complement other omics approaches, such as genomics and proteomics. By comparing metabolomics to other diagnostic modalities, the study underscores its unparalleled sensitivity to subtle metabolic alterations, establishing it as an indispensable component of non-invasive cancer detection frameworks. Additionally, the review emphasizes the practical application of metabolomics in developing non-invasive diagnostic tools and its integration with computational models, such as machine learning algorithms, which has not been extensively explored in existing literature. Further research and technological advancements are warranted to refine metabolomic strategies, optimize biomarker panels, and facilitate their translation into routine clinical practice. Embracing metabolomics as an integral component of cancer screening protocols holds immense potential to revolutionize early detection strategies, ultimately improving patient outcomes through timely interventions and personalized therapeutic approaches.

## Figures and Tables

**Table 1:** Routine methods for diagnosing cancers.

Diagnostic Method	Current Methods	Accuracy	Predictive Value	Costs (Approx.)	False Positives/ Negatives	Ref
Imaging techniques	X-rays	Moderate (~60 %)	High for detecting fractures/tumors	Low	High false positives for benign masses	[5]
	Computed tomography	High (~85–95 %)	Excellent for soft tissue tumors	Medium	False positives in inflammation	[5]
	Magnetic resonance imaging	High (~90–95 %)	Precise for brain, soft tissue tumors	High	Low false positives, but overdiagnosis	[5]
	Positron emission tomography	High (~90 %)	Effective for metabolic activity	Very high	False positives in infections	[5,6]
	Ultrasound	Variable (~50–80 %)	High for fluid-filled structures	Low	High false negatives in dense tissues	[5,6]
Biopsy	Fine needle aspiration	High (~85 %)	Cost-effective for small masses	Low	False negatives in deeper tissues	[7,8]
	Core needle biopsy	Very high (~95 %)	Accurate for solid tumors	Medium	Low false negatives	[8]
	Surgical biopsy	Very high (~98 %)	Gold standard for definitive diagnosis	High	Very low false negatives	[8]
Blood tests	Tumor marker tests	Variable (~50–70 %)	Useful for monitoring	Low	High false positives for non-specific markers	[9]
Genomic testing	Next-generation sequencing (NGS)	Very high (~99 %)	Excellent for identifying mutations	Very high	Low false negatives; rare contamination	[10,11]
	Polymerase chain reaction (PCR)	Very high (~98 %)	Precise for detecting specific genes	Medium	Low false negatives	[11]
	Fluorescence in situ hybridization (FISH)	Very high (~97 %)	Effective for chromosomal abnormalities	High	Low false positives	[11]
Endoscopy	Upper gastrointestinal endoscopy	High (~90 %)	Detects lesions, polyps	High	Low false negatives	[12]
	Colonoscopy	Very high (~95 %)	Highly accurate for colon cancer	High	Very low false positives	[12]
Pathology	Histopathology	Very high (~98 %)	Gold standard for confirming cancer	High	Rare false negatives	[13]
Liquid biopsy	Circulating tumor cells (CTCs)	High (~85–90 %)	Non-invasive; monitors metastasis	Medium	High false negatives in early stages	[14]

**Table 2:** Various omics approaches used in the early diagnosis of cancer.

Omics Approach	Definition	Application in Cancer Diagnosis	Common Techniques	Advantages	Disadvantages	Ref
Genomics	Study of an organism’s entire DNA, including genes.	Identifying genetic mutations and variations associated with cancer.	Next-generation sequencing	High sensitivity to genetic variations; essential for precision medicine.	Expensive; requires high-quality DNA; limited insight into non-genetic cancer drivers.	[17]
Transcriptomics	Study of RNA molecules, including mRNA, miRNA, and non-coding RNA.	Assessing gene expression patterns in cancer cells.	Microarray, RNA-Seq	Captures dynamic gene expression; identifies functional changes in cancer.	Sensitive to RNA degradation; complex data analysis; variability between samples.	[18]
Proteomics	Study of the entire set of proteins expressed by a cell.	Identifying protein biomarkers associated with cancer.	Mass spectrometry, protein microarrays	Direct insight into functional molecules; identifies post-translational modifications.	Challenging sample preparation; limited coverage of low-abundance proteins.	[19]
Metabolomics	Study of small molecules (metabolites) involved in cellular processes.	Profiling the metabolic signature of cancer cells.	Mass spectrometry, nuclear magnetic resonance	High sensitivity to early metabolic changes; non-invasive sample collection; supports personalized medicine.	Complex data analysis; highly influenced by external factors like diet or environment; requires advanced tools.	[20,21]
Epigenomics	Study of epigenetic modifications, such as DNA methylation.	Identifying epigenetic alterations associated with cancer.	Bisulfite sequencing, chromatin immunoprecipitation	Explains gene regulation beyond genetic mutations; reversible nature aids therapeutic targeting.	Epigenetic changes are tissue-specific; requires careful sample handling to avoid degradation.	[20]
Glycomics	Study of the structure and function of carbohydrates (glycans).	Analyzing changes in glycosylation patterns in cancer cells.	Mass spectrometry, liquid chromatography	Identifies unique glycosylation patterns in cancer; potential for therapeutic targeting.	Glycan analysis is complex and time-consuming; lacks standardized workflows.	[22]
Lipidomics	Study of lipid molecules and their role in cellular functions.	Profiling changes in lipid composition associated with cancer.	Mass spectrometry, gas chromatography	Highlights changes in energy storage and signalling; critical for understanding cancer cell metabolism.	Requires specialized techniques for lipid isolation and quantification; prone to contamination.	[23]
Immunomics	Study of the immune system, including immune cells and their interactions.	Analyzing the immune response to cancer cells.	Immune profiling, flow cytometry	Provides insight into cancer-immune system interactions; supports immunotherapy development.	Complex immune networks make data interpretation challenging; subject to variability between individuals.	[24–26]

**Table 3:** Technological advancements in metabolomics for the timely diagnosis of cancer.

Technological Advancements	Description	Ref
Early detection	Enables early detection of abnormal metabolic patterns, allowing cancer diagnosis at treatable stages and improving survival rates.	[39]
Integration with multi-omics	Combines metabolomics with other omics data (genomics, proteomics) to provide a comprehensive understanding of cancer biology and diagnostics.	[40]
Machine learning applications	Integrates machine learning algorithms with metabolomics for improved biomarker discovery and enhanced diagnostic accuracy across cancer types.	[32,34,41]
Non-invasive diagnostic tools	Advances in metabolomics enable the development of non-invasive tests (e.g., blood, urine, and saliva analysis) for cancer detection.	[34,35,42]
Metabolomics	Identifies metabolic changes associated with cancer, offering insights into disease progression and aiding in developing diagnostic strategies.	[39]
Biomarker discovery	Detects potential biomarkers for specific cancers, leading to the development of diagnostic tools for non-invasive and early detection.	[40,43]
Precision oncology	Uses metabolomics data to tailor medications based on the unique metabolic traits of each patient’s cancer, enhancing treatment specificity.	[40]
Personalized medicine	Facilitates the creation of tailored treatment plans by analyzing individual metabolic profiles, enabling targeted therapies for specific cancers.	[44]
Treatment monitoring	Tracks metabolic changes in response to therapies, enabling adjustments for more effective treatments and better patient outcomes.	[45]

**Table 4:** Differences in metabolic activity between healthy cells and cancer cells.

Feature	Healthy Cell Metabolism	Cancer Cell Metabolism	References
Energy source preference	Utilizes glucose oxidation (aerobic) efficiently via the citric acid cycle for ATP production.	Primarily engages in aerobic glycolysis (Warburg effect), converting glucose to lactate despite oxygen availability.	[46,49]
Oxygen dependence	Strictly oxygen-dependent for energy production.	Facultative anaerobes that can function with or without oxygen.	[44,47]
Lactate production	Minimal lactate production under normal physiological conditions.	Elevated lactate production occurs even in the presence of oxygen.	[44,47]
Metabolic adaptation	Flexible metabolism, adjusting to various nutrient sources (glucose, fats, amino acids) based on availability.	Over-reliance on glucose, often neglecting other available nutrients.	[44,46,50]
Macromolecule synthesis	Balanced synthesis of proteins, lipids, and nucleic acids essential for cell maintenance and function.	Prioritizes the synthesis of macromolecules related to growth and division, often compromising other vital processes.	[44,46]
Regulation	Tightly regulated by growth factors and signalling pathways to maintain homeostasis.	Deregulated metabolism often driven by mutations in oncogenes and tumor suppressor genes.	[49,50]
Energy efficiency	High efficiency in ATP production through oxidative phosphorylation.	Lower efficiency due to reliance on aerobic glycolysis, necessitating increased glucose uptake for energy.	[39,44]
Integration with multi-omics	Healthy cells maintain metabolic processes in alignment with normal physiological pathways, showing minimal interaction with other omics layers (e.g., genomics, proteomics).	Cancer cells exhibit extensive crosstalk with multi-omics layers, integrating metabolomics with altered gene expression and protein regulation for tumor progression.	[39,40,53]
Metabolomic biomarkers	Healthy cells do not produce significant levels of cancer-associated metabolites.	Cancer cells produce unique metabolic biomarkers (e.g., 2-hydroxyglutarate) that are pivotal in early diagnosis and treatment monitoring.	[45,53]

**Table 5:** Comparison of targeted and untargeted metabolomics approaches.

Feature	Targeted Metabolomics	Untargeted Metabolomics	References
Scope	Narrow focus on predefined metabolites.	Broad analysis of the entire metabolome.	[57]
Sensitivity	High sensitivity for specific metabolites.	May have lower sensitivity due to broader scope.	[59]
Specificity	High specificity for known biomarkers.	Identifies a wide range of metabolites, including unexpected and novel biomarkers.	[55]
Data analysis	Straightforward analysis focusing on a specific set of metabolites.	Complex analysis requiring advanced statistical methods.	[57]
Quantification	Accurate quantification of known metabolites.	Challenges in quantification, especially for unidentified metabolites.	[31,59,60]
Applications	Commonly used for validation, pathway analysis, and targeted biomarker discovery.	Valuable for exploratory studies, biomarker discovery, and providing a holistic view of the metabolome.	[57,59,61]
